# Tris[tris­(1,10-phenanthroline-κ^2^ *N*,*N*′)iron(II)] dodeca­tungstoferrate dihydrate

**DOI:** 10.1107/S1600536808009896

**Published:** 2008-04-16

**Authors:** Feng-Xia Ma, Ya-Guang Chen, Dong-Mei Shi

**Affiliations:** a Department of Chemistry, Northeast Normal University, Changchun 130024, People’s Republic of China; b Basic Science Department, Jilin Agricultural Science and Technology College, Jilin 132101, People’s Republic of China

## Abstract

The title compound, [Fe(C_12_H_8_N_2_)_3_]_3_[FeW_12_O_40_]·2H_2_O, was prepared under hydro­thermal conditions. The discrete Keggin-type [FeW_12_O_40_]^6−^ heteropolyoxoanion has threefold symmetry, with the Fe^II^ atom located on the threefold rotation axis. The central FeO_4_ tetra­hedron in the anion shares its O atoms with four W_3_O_13_ trinuclear units, each of which is made up of three edge-shared WO_6_ octa­hedral units. The Fe^II^ atom in the complex cation, *viz *[Fe(phen)_3_]^2+^ (phen is 1,10-phen­anthroline), shows a slightly distorted octa­hedral geometry defined by six N atoms from three phen ligands. The polyoxoanions pack together with the cations, with the disordered water mol­ecules located in voids; the site occupancy factor for each water O atom is 0.33.

## Related literature

For related literature, see: Brown (2002 ▶); Misono (1987 ▶); Pope (1983 ▶).
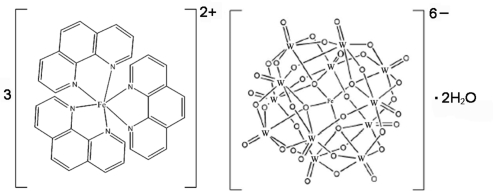


## Experimental

### 

#### Crystal data

[Fe(C_12_H_8_N_2_)_3_]_3_[FeW_12_O_40_]·2H_2_O
*M* *_r_* = 4727.47
Trigonal, 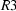

*a* = 25.088 (5) Å
*c* = 17.231 (5) Å
*V* = 9392 (4) Å^3^
*Z* = 3
Mo *K*α radiation
μ = 11.51 mm^−1^
*T* = 293 (2) K
0.24 × 0.21 × 0.20 mm

#### Data collection

Bruker SMART APEX CCD area-detector diffractometer
Absorption correction: multi-scan (*SADABS*; Sheldrick, 1996 ▶) *T*_min_ = 0.075, *T*_max_ = 0.104
16084 measured reflections
6154 independent reflections
5557 reflections with *I* > 2σ(*I*)
*R*_int_ = 0.034

#### Refinement

*R*[*F* ^2^ > 2σ(*F* ^2^)] = 0.035
*wR*(*F* ^2^) = 0.093
*S* = 1.03
6154 reflections
560 parameters
1 restraint
H-atom parameters constrained
Δρ_max_ = 1.62 e Å^−3^
Δρ_min_ = −1.55 e Å^−3^
Absolute structure: Flack (1983 ▶), 3713 Friedel pairs
Flack parameter: −0.006 (6)

### 

Data collection: *SMART* (Bruker, 2007 ▶); cell refinement: *SAINT* (Bruker, 2007 ▶); data reduction: *SAINT*; program(s) used to solve structure: *SHELXS97* (Sheldrick, 2008 ▶); program(s) used to refine structure: *SHELXL97* (Sheldrick, 2008 ▶); molecular graphics: *SHELXTL-Plus* (Sheldrick, 2008 ▶); software used to prepare material for publication: *SHELXL97*.

## Supplementary Material

Crystal structure: contains datablocks global, I. DOI: 10.1107/S1600536808009896/hy2126sup1.cif

Structure factors: contains datablocks I. DOI: 10.1107/S1600536808009896/hy2126Isup2.hkl

Additional supplementary materials:  crystallographic information; 3D view; checkCIF report

## Figures and Tables

**Table 1:** Selected bond lengths (Å)

Fe1—O13	1.826 (8)
Fe1—O1	1.833 (18)
Fe2—N6	1.969 (11)
Fe2—N3	1.971 (12)
Fe2—N1	1.972 (10)
Fe2—N5	1.979 (12)
Fe2—N2	1.986 (11)
Fe2—N4	2.001 (11)
W1—O3	1.692 (9)
W1—O4	1.898 (9)
W1—O7	1.943 (8)
W1—O12	1.950 (8)
W1—O1	2.230 (9)
W2—O5	1.702 (10)
W2—O7	1.858 (9)
W2—O9	1.940 (9)
W2—O14^i^	1.954 (9)
W2—O11^i^	1.963 (10)
W2—O13^i^	2.211 (8)
W3—O6	1.742 (11)
W3—O2^i^	1.903 (9)
W3—O2	1.911 (9)
W3—O11	1.951 (10)
W3—O10	1.961 (10)
W3—O13	2.229 (10)
W4—O8	1.678 (9)
W4—O9	1.896 (8)
W4—O4	1.904 (9)
W4—O10	1.944 (11)
W4—O14	2.001 (9)
W4—O13	2.221 (8)

Symmetry code: (i) 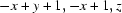.

**Fig. 1.**
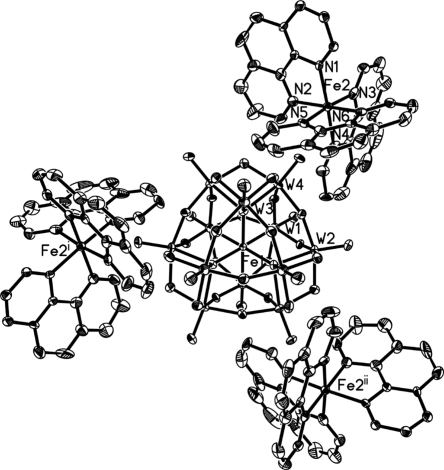
The structure of the title compound. Displacement ellipsoids are drawn at the 30% probability level. H atoms and disordered water molecules are omitted for clarity. [Symmetry codes: (i) -y + 1, x-y, z; (ii) -x + y+1, -x + 1, z.]

## supplementary crystallographic information

### Comment

Polyoxometalates (POMs) have attracted attention in recent years, not only because of their structural diversity but also because of their potential applications in medicine, material science, especially in catalysis (Misono, 1987; Pope, 1983).

The structure of the title compound is built up from three complex cations, [Fe(phen)_3_]^2+^ (phen = 1,10-phenanthroline), one Keggin-type anion, [FeW_12_O_40_]^6-^, and two disordered water molecules. The heteropolyoxoanion [FeW_12_O_40_]^6-^ has threefold symmetry with the Fe1 atom located on the threefold rotation axis. The Fe2 atom in the cation is coordinated by six N atoms from three phen molecules, forming a distorted FeN_6_ octahedron with Fe—N bond distances ranging from 1.969 (11) to 2.001 (11)Å (Fig. 1; Table 1).

Results of bond valence sum (BVS) calculations (Brown, 2002) are in accordance with expected values for hexavalent tungsten (average 6.15 valence units for the 12 W atoms) and divalent iron (1.92 valence units for the Fe atom in the complex cation).

### Experimental

A mixture of ammonium tungstate monohydrate (1.005 g, 0.6 mmol), FeCl_3_.6H_2_O (0.157 g, 0.6 mmol), phen.H_2_O (0.256 g, 1.3 mmol), NH_4_VO_3_(0.053 g, 0.5 mmol), oxalic acid dihydrate(0.205 g, 1.6 mmol) and H_2_O (10 ml) was adjusted to pH = 5.8 by addition of 2 mol *L*^-1^ NaOH under stirring for 30 min. The final solution was transferred into a 25 ml Teflon-lined autoclave and was heated at 453 K for 96 h. Then the autoclave was cooled in a rate of 10 K h^-1^ to room temperature. Red block-like crystals were filtered off, washed with distilled water, and dried at ambient temperature (40% yield on W).

### Refinement

H atoms on C atoms were positioned geometrically and refined as riding atoms, with C—H = 0.93 Å and *U*_iso_(H) = 1.2*U*_eq_(C). H atoms of the disordered water molecules were not located. In the final difference Fourier map, the highest peak is 2.83Å away from O2W and the deepest hole is 0.23 Å from Fe1.

### Crystal data

**Table:**

[Fe(C_12_H_8_N_2_)_3_]_3_[FeW_12_O_40_]·2H_2_O	*Z* = 3
*M**_r_* = 4727.47	*F*_000_ = 6593.7
Trigonal, *R*3	*D*_x_ = 2.527 Mg m^−^^3^
Hall symbol: R 3	Mo *K*α radiation λ = 0.71073 Å
*a* = 25.088 (5) Å	Cell parameters from 5571 reflections
*b* = 25.088 (5) Å	θ = 1.5–25.1º
*c* = 17.231 (5) Å	µ = 11.51 mm^−^^1^
α = 90º	*T* = 293 (2) K
β = 90º	Block, red
γ = 120º	0.24 × 0.21 × 0.20 mm
*V* = 9392 (4) Å^3^	

### Data collection

**Table:**

Bruker SMART APEX CCD area-detector diffractometer	6154 independent reflections
Radiation source: fine-focus sealed tube	5557 reflections with *I* > 2σ(*I*)
Monochromator: graphite	*R*_int_ = 0.034
*T* = 293(2) K	θ_max_ = 25.1º
φ and ω scans	θ_min_ = 1.5º
Absorption correction: multi-scan(SADABS; Sheldrick, 1996)	*h* = −29→29
*T*_min_ = 0.075, *T*_max_ = 0.104	*k* = −29→29
16084 measured reflections	*l* = −20→12

### Refinement

**Table:**

Refinement on *F*^2^	Hydrogen site location: inferred from neighbouring sites
Least-squares matrix: full	H-atom parameters constrained
*R*[*F*^2^ > 2σ(*F*^2^)] = 0.035	*w* = 1/[σ^2^(*F*_o_^2^) + (0.0566*P*)^2^ + 7.5378*P*] where *P* = (*F*_o_^2^ + 2*F*_c_^2^)/3
*wR*(*F*^2^) = 0.093	(Δ/σ)_max_ = 0.001
*S* = 1.04	Δρ_max_ = 1.62 e Å^−^^3^
6154 reflections	Δρ_min_ = −1.55 e Å^−^^3^
560 parameters	Extinction correction: none
1 restraint	Absolute structure: Flack (1983), 3713 Friedel pairs
Primary atom site location: structure-invariant direct methods	Flack parameter: −0.006 (6)
Secondary atom site location: difference Fourier map	

### Fractional atomic coordinates and isotropic or equivalent isotropic displacement parameters (Å^2^)

**Table:**

	*x*	*y*	*z*	*U*_iso_*/*U*_eq_	Occ. (<1)
Fe1	0.6667	0.3333	0.3946 (2)	0.0268 (6)	
Fe2	0.32586 (8)	0.15648 (8)	0.72949 (11)	0.0297 (4)	
W1	0.58806 (3)	0.33016 (3)	0.22372 (4)	0.03811 (16)	
W2	0.58842 (3)	0.41729 (3)	0.39586 (3)	0.04278 (17)	
W3	0.58253 (3)	0.24702 (3)	0.55437 (4)	0.04819 (18)	
W4	0.50462 (2)	0.24669 (3)	0.39934 (3)	0.04051 (16)	
C1	0.2245 (6)	0.0581 (6)	0.8268 (8)	0.035 (3)	
H1A	0.2201	0.0887	0.8513	0.043*	
C2	0.1869 (6)	−0.0018 (6)	0.8499 (9)	0.040 (3)	
H2A	0.1596	−0.0099	0.8907	0.048*	
C3	0.1885 (6)	−0.0479 (6)	0.8161 (8)	0.042 (3)	
H3A	0.1614	−0.0885	0.8298	0.050*	
C4	0.2355 (6)	−0.0320 (5)	0.7551 (8)	0.035 (3)	
C5	0.2712 (5)	0.0293 (5)	0.7356 (8)	0.035 (3)	
C6	0.3150 (6)	0.0474 (6)	0.6755 (8)	0.044 (3)	
C7	0.3259 (7)	0.0014 (6)	0.6414 (9)	0.052 (4)	
C8	0.2083 (6)	0.1176 (7)	0.6442 (9)	0.048 (4)	
H8A	0.1871	0.0856	0.6792	0.057*	
C9	0.1760 (9)	0.1237 (9)	0.5801 (13)	0.079 (7)	
H9A	0.1338	0.0965	0.5779	0.095*	
C10	0.1990 (11)	0.1623 (11)	0.5263 (12)	0.080 (6)	
H10A	0.1757	0.1626	0.4846	0.096*	
C11	0.3893 (7)	0.1268 (7)	0.6061 (10)	0.062 (5)	
H11A	0.4110	0.1683	0.5931	0.074*	
C12	0.4027 (12)	0.0851 (9)	0.5665 (12)	0.095 (8)	
H12A	0.4323	0.1003	0.5275	0.114*	
C13	0.2444 (7)	−0.0784 (7)	0.7168 (11)	0.063 (5)	
H13A	0.2185	−0.1201	0.7264	0.076*	
C14	0.4425 (7)	0.2691 (7)	0.6851 (10)	0.054 (4)	
H14A	0.4602	0.2610	0.7277	0.065*	
C15	0.2629 (13)	0.2054 (10)	0.5327 (10)	0.085 (7)	
C16	0.3002 (18)	0.2552 (15)	0.4770 (12)	0.140 (14)	
H16A	0.2817	0.2599	0.4327	0.168*	
C17	0.3649 (14)	0.2965 (12)	0.4905 (13)	0.094 (8)	
H17A	0.3881	0.3268	0.4542	0.113*	
C18	0.3929 (12)	0.2912 (8)	0.5584 (11)	0.081 (7)	
C19	0.3567 (8)	0.2426 (6)	0.6103 (9)	0.051 (4)	
C20	0.3430 (5)	0.2139 (5)	0.8739 (7)	0.033 (3)	
C21	0.3882 (6)	0.1979 (6)	0.8721 (7)	0.038 (3)	
C22	0.4287 (7)	0.2118 (7)	0.9329 (9)	0.054 (4)	
C23	0.4718 (8)	0.1912 (9)	0.9277 (12)	0.070 (6)	
H23A	0.5000	0.1999	0.9675	0.084*	
C24	0.4291 (8)	0.1453 (9)	0.8025 (11)	0.072 (5)	
H24A	0.4292	0.1232	0.7592	0.086*	
C25	0.2933 (11)	−0.0580 (8)	0.6650 (11)	0.081 (7)	
H25A	0.3040	−0.0861	0.6460	0.097*	
C26	0.2600 (6)	0.2131 (6)	0.8112 (9)	0.042 (3)	
H26A	0.2338	0.2023	0.7687	0.051*	
C27	0.2488 (7)	0.2413 (7)	0.8753 (9)	0.045 (4)	
H27A	0.2156	0.2482	0.8747	0.054*	
C28	0.2852 (8)	0.2575 (7)	0.9352 (9)	0.059 (5)	
H28A	0.2784	0.2772	0.9765	0.071*	
C29	0.3371 (7)	0.2455 (6)	0.9392 (8)	0.045 (3)	
C30	0.3740 (9)	0.2556 (8)	0.9998 (10)	0.068 (5)	
H30A	0.3661	0.2705	1.0452	0.081*	
C31	0.4232 (8)	0.2451 (7)	0.9995 (9)	0.063 (5)	
H31A	0.4516	0.2587	1.0399	0.076*	
C32	0.2986 (8)	0.2029 (6)	0.5974 (9)	0.054 (4)	
C33	0.4560 (12)	0.3308 (9)	0.5740 (13)	0.088 (8)	
H33A	0.4795	0.3637	0.5411	0.105*	
C34	0.3739 (9)	0.0237 (9)	0.5835 (11)	0.083 (6)	
H34A	0.3847	−0.0025	0.5589	0.100*	
C35	0.4820 (8)	0.3208 (7)	0.6360 (13)	0.070 (6)	
H35A	0.5236	0.3461	0.6471	0.084*	
C36	0.4723 (8)	0.1583 (10)	0.8642 (13)	0.084 (6)	
H36A	0.5005	0.1446	0.8613	0.101*	
N1	0.2665 (4)	0.0749 (4)	0.7718 (6)	0.027 (2)	
N2	0.3485 (5)	0.1086 (5)	0.6585 (7)	0.040 (3)	
N3	0.2656 (6)	0.1551 (5)	0.6549 (6)	0.040 (3)	
N4	0.3847 (6)	0.2337 (5)	0.6735 (7)	0.041 (3)	
N5	0.3875 (5)	0.1656 (5)	0.8079 (7)	0.040 (3)	
N6	0.3062 (5)	0.2008 (5)	0.8083 (6)	0.031 (2)	
O1	0.6667	0.3333	0.2882 (10)	0.038 (4)	
O2	0.6684 (4)	0.2746 (4)	0.5635 (6)	0.047 (2)	
O3	0.5416 (5)	0.3302 (5)	0.1526 (6)	0.046 (2)	
O4	0.5342 (4)	0.2705 (4)	0.2961 (5)	0.040 (2)	
O5	0.5375 (5)	0.4431 (5)	0.3877 (7)	0.059 (3)	
O6	0.5548 (5)	0.2172 (6)	0.6463 (6)	0.063 (3)	
O7	0.5918 (4)	0.3915 (4)	0.2958 (5)	0.037 (2)	
O8	0.4282 (4)	0.2170 (5)	0.3920 (7)	0.056 (3)	
O9	0.5345 (4)	0.3311 (4)	0.4206 (5)	0.040 (2)	
O10	0.5036 (4)	0.2295 (4)	0.5095 (6)	0.049 (3)	
O11	0.5687 (4)	0.1702 (4)	0.5081 (6)	0.046 (2)	
O12	0.6053 (4)	0.2694 (4)	0.1776 (6)	0.040 (2)	
O13	0.5982 (4)	0.2633 (4)	0.4271 (6)	0.036 (2)	
O14	0.5038 (4)	0.1676 (4)	0.3806 (6)	0.045 (2)	
O1W	0.5848 (13)	0.3187 (15)	0.7626 (19)	0.053 (9)	0.33
O2W	0.5964 (9)	0.2622 (9)	1.0192 (12)	0.013 (4)*	0.33

### Atomic displacement parameters (Å^2^)

**Table:**

	*U*^11^	*U*^22^	*U*^33^	*U*^12^	*U*^13^	*U*^23^
Fe1	0.0211 (7)	0.0211 (7)	0.0382 (16)	0.0105 (4)	0.000	0.000
Fe2	0.0294 (9)	0.0271 (10)	0.0303 (9)	0.0123 (8)	0.0027 (7)	−0.0021 (8)
W1	0.0322 (3)	0.0341 (3)	0.0487 (4)	0.0171 (2)	−0.0097 (3)	−0.0013 (3)
W2	0.0389 (3)	0.0400 (3)	0.0606 (5)	0.0282 (3)	−0.0024 (3)	−0.0043 (3)
W3	0.0459 (4)	0.0465 (4)	0.0512 (4)	0.0223 (3)	0.0143 (3)	0.0129 (3)
W4	0.0216 (3)	0.0361 (3)	0.0588 (4)	0.0107 (2)	0.0040 (2)	−0.0005 (3)
C1	0.038 (7)	0.033 (7)	0.039 (8)	0.020 (6)	0.012 (6)	0.008 (6)
C2	0.042 (8)	0.042 (8)	0.042 (9)	0.025 (7)	0.007 (6)	0.009 (7)
C3	0.038 (7)	0.035 (7)	0.055 (9)	0.020 (6)	−0.008 (6)	0.006 (6)
C4	0.039 (7)	0.020 (6)	0.047 (8)	0.016 (5)	0.005 (6)	0.000 (5)
C5	0.029 (6)	0.028 (7)	0.050 (8)	0.016 (5)	−0.003 (6)	0.001 (6)
C6	0.052 (8)	0.047 (8)	0.037 (8)	0.029 (7)	0.004 (7)	−0.020 (6)
C7	0.060 (9)	0.040 (8)	0.051 (10)	0.020 (7)	0.014 (7)	−0.008 (7)
C8	0.038 (8)	0.040 (7)	0.058 (10)	0.013 (6)	−0.024 (7)	−0.011 (6)
C9	0.082 (13)	0.064 (12)	0.106 (17)	0.048 (11)	−0.068 (13)	−0.046 (12)
C10	0.094 (16)	0.082 (15)	0.067 (14)	0.046 (13)	−0.038 (12)	−0.005 (11)
C11	0.061 (10)	0.033 (8)	0.082 (13)	0.017 (7)	0.034 (9)	0.010 (8)
C12	0.15 (2)	0.066 (13)	0.075 (15)	0.056 (14)	0.066 (15)	−0.001 (10)
C13	0.052 (10)	0.042 (9)	0.083 (13)	0.014 (8)	0.012 (9)	−0.012 (8)
C14	0.050 (9)	0.045 (9)	0.055 (10)	0.014 (8)	0.016 (8)	−0.010 (7)
C15	0.18 (2)	0.085 (14)	0.035 (10)	0.097 (17)	0.013 (12)	0.019 (9)
C16	0.31 (4)	0.18 (3)	0.044 (14)	0.21 (3)	0.06 (2)	0.056 (17)
C17	0.16 (2)	0.101 (19)	0.069 (16)	0.102 (19)	0.033 (16)	0.041 (13)
C18	0.15 (2)	0.056 (11)	0.056 (12)	0.067 (13)	0.049 (12)	0.015 (9)
C19	0.081 (12)	0.036 (8)	0.042 (9)	0.033 (8)	0.035 (8)	0.013 (7)
C20	0.028 (6)	0.023 (6)	0.035 (7)	0.003 (5)	0.005 (5)	0.006 (5)
C21	0.033 (7)	0.047 (8)	0.030 (7)	0.017 (6)	−0.010 (6)	−0.002 (6)
C22	0.043 (8)	0.045 (9)	0.058 (11)	0.009 (7)	0.001 (7)	0.010 (7)
C23	0.037 (9)	0.085 (13)	0.074 (14)	0.020 (9)	−0.029 (9)	0.010 (11)
C24	0.046 (9)	0.089 (13)	0.088 (14)	0.040 (10)	−0.014 (9)	−0.009 (11)
C25	0.139 (18)	0.048 (10)	0.075 (13)	0.062 (12)	0.032 (13)	−0.011 (9)
C26	0.030 (7)	0.035 (7)	0.056 (10)	0.012 (6)	−0.005 (6)	0.001 (6)
C27	0.055 (9)	0.055 (9)	0.037 (9)	0.035 (8)	−0.001 (7)	−0.006 (7)
C28	0.093 (13)	0.050 (9)	0.039 (10)	0.040 (9)	0.025 (9)	−0.008 (7)
C29	0.052 (9)	0.043 (8)	0.033 (8)	0.019 (7)	−0.011 (7)	−0.018 (6)
C30	0.085 (13)	0.059 (11)	0.059 (12)	0.036 (10)	−0.018 (10)	−0.021 (8)
C31	0.073 (12)	0.040 (8)	0.046 (10)	0.005 (8)	−0.026 (9)	−0.006 (7)
C32	0.090 (12)	0.030 (8)	0.047 (10)	0.034 (8)	0.019 (8)	0.007 (7)
C33	0.117 (19)	0.051 (11)	0.077 (16)	0.029 (13)	0.071 (14)	0.014 (11)
C34	0.083 (14)	0.073 (13)	0.095 (15)	0.039 (11)	0.026 (12)	−0.030 (11)
C35	0.046 (10)	0.035 (9)	0.101 (17)	0.000 (8)	0.047 (11)	0.002 (9)
C36	0.050 (11)	0.116 (17)	0.101 (18)	0.052 (12)	−0.006 (10)	0.006 (14)
N1	0.031 (5)	0.027 (5)	0.028 (6)	0.019 (5)	0.001 (4)	0.000 (4)
N2	0.041 (7)	0.039 (6)	0.037 (7)	0.019 (5)	0.009 (5)	−0.004 (5)
N3	0.061 (8)	0.039 (6)	0.028 (6)	0.031 (6)	−0.001 (6)	−0.006 (5)
N4	0.063 (8)	0.022 (5)	0.031 (7)	0.016 (6)	0.023 (6)	0.000 (5)
N5	0.023 (5)	0.037 (6)	0.049 (7)	0.008 (5)	0.004 (5)	−0.001 (5)
N6	0.030 (6)	0.026 (5)	0.024 (6)	0.006 (5)	−0.002 (4)	−0.003 (4)
O1	0.030 (5)	0.030 (5)	0.055 (11)	0.015 (2)	0.000	0.000
O2	0.046 (6)	0.043 (6)	0.054 (7)	0.025 (5)	0.003 (5)	0.008 (5)
O3	0.048 (6)	0.048 (6)	0.048 (6)	0.028 (5)	−0.022 (5)	−0.008 (5)
O4	0.040 (5)	0.028 (5)	0.049 (6)	0.013 (4)	0.005 (4)	−0.003 (4)
O5	0.054 (6)	0.038 (6)	0.096 (9)	0.030 (5)	−0.002 (6)	−0.010 (5)
O6	0.062 (7)	0.072 (8)	0.056 (8)	0.035 (6)	0.016 (6)	0.015 (6)
O7	0.041 (5)	0.028 (5)	0.050 (6)	0.022 (4)	−0.007 (4)	−0.009 (4)
O8	0.025 (5)	0.045 (6)	0.089 (9)	0.010 (4)	0.013 (5)	−0.003 (6)
O9	0.040 (5)	0.032 (5)	0.053 (6)	0.021 (4)	0.006 (4)	−0.002 (4)
O10	0.049 (6)	0.033 (5)	0.054 (7)	0.013 (5)	0.020 (5)	−0.004 (4)
O11	0.046 (6)	0.041 (5)	0.054 (7)	0.024 (5)	0.005 (5)	0.015 (5)
O12	0.034 (5)	0.032 (5)	0.054 (7)	0.016 (4)	0.000 (4)	0.001 (4)
O13	0.036 (5)	0.026 (4)	0.044 (6)	0.013 (4)	0.002 (4)	0.002 (4)
O14	0.039 (5)	0.041 (5)	0.058 (7)	0.022 (5)	0.003 (4)	−0.005 (4)
O1W	0.048 (18)	0.08 (2)	0.05 (2)	0.041 (17)	−0.010 (14)	−0.042 (16)

### Geometric parameters (Å, °)

**Table:**

Fe1—O13^i^	1.826 (8)	C11—C12	1.42 (2)
Fe1—O13	1.826 (8)	C11—H11A	0.9300
Fe1—O13^ii^	1.826 (8)	C12—C34	1.37 (2)
Fe1—O1	1.833 (18)	C12—H12A	0.9300
Fe2—N6	1.969 (11)	C13—C25	1.39 (2)
Fe2—N3	1.971 (12)	C13—H13A	0.9300
Fe2—N1	1.972 (10)	C14—N4	1.283 (19)
Fe2—N5	1.979 (12)	C14—C35	1.45 (2)
Fe2—N2	1.986 (11)	C14—H14A	0.9300
Fe2—N4	2.001 (11)	C15—C32	1.45 (3)
W1—O3	1.692 (9)	C15—C16	1.48 (3)
W1—O4	1.898 (9)	C16—C17	1.44 (4)
W1—O7	1.943 (8)	C16—H16A	0.9300
W1—O12^ii^	1.950 (9)	C17—C18	1.41 (3)
W1—O12	1.950 (8)	C17—H17A	0.9300
W1—O1	2.230 (9)	C18—C33	1.41 (3)
W2—O5	1.702 (10)	C18—C19	1.42 (2)
W2—O7	1.858 (9)	C19—C32	1.31 (2)
W2—O9	1.940 (9)	C19—N4	1.37 (2)
W2—O14^ii^	1.954 (9)	C20—C21	1.380 (17)
W2—O11^ii^	1.963 (10)	C20—N6	1.390 (16)
W2—O13^ii^	2.211 (8)	C20—C29	1.425 (18)
W3—O6	1.742 (11)	C21—N5	1.367 (17)
W3—O2^ii^	1.903 (9)	C21—C22	1.377 (19)
W3—O2	1.911 (9)	C22—C23	1.42 (2)
W3—O11	1.951 (10)	C22—C31	1.47 (2)
W3—O10	1.961 (10)	C23—C36	1.37 (3)
W3—O13	2.229 (10)	C23—H23A	0.9300
W4—O8	1.678 (9)	C24—N5	1.373 (19)
W4—O9	1.896 (8)	C24—C36	1.44 (2)
W4—O4	1.904 (9)	C24—H24A	0.9300
W4—O10	1.944 (11)	C25—H25A	0.9300
W4—O14	2.001 (9)	C26—N6	1.341 (16)
W4—O13	2.221 (8)	C26—C27	1.41 (2)
C1—N1	1.319 (15)	C26—H26A	0.9300
C1—C2	1.375 (18)	C27—C28	1.30 (2)
C1—H1A	0.9300	C27—H27A	0.9300
C2—C3	1.313 (18)	C28—C29	1.48 (2)
C2—H2A	0.9300	C28—H28A	0.9300
C3—C4	1.477 (18)	C29—C30	1.33 (2)
C3—H3A	0.9300	C30—C31	1.39 (2)
C4—C5	1.378 (16)	C30—H30A	0.9300
C4—C13	1.451 (18)	C31—H31A	0.9300
C5—N1	1.360 (15)	C32—N3	1.455 (18)
C5—C6	1.409 (18)	C33—C35	1.34 (3)
C6—N2	1.363 (17)	C33—H33A	0.9300
C6—C7	1.440 (18)	C34—H34A	0.9300
C7—C25	1.36 (2)	C35—H35A	0.9300
C7—C34	1.44 (2)	C36—H36A	0.9300
C8—N3	1.279 (17)	O1—W1^i^	2.230 (9)
C8—C9	1.42 (2)	O1—W1^ii^	2.230 (9)
C8—H8A	0.9300	O2—W3^i^	1.903 (9)
C9—C10	1.25 (3)	O11—W2^i^	1.963 (10)
C9—H9A	0.9300	O12—W1^i^	1.950 (8)
C10—C15	1.42 (3)	O13—W2^i^	2.211 (8)
C10—H10A	0.9300	O14—W2^i^	1.954 (9)
C11—N2	1.267 (17)		
			
O13^i^—Fe1—O13	111.0 (3)	C34—C12—C11	122.6 (17)
O13^i^—Fe1—O13^ii^	111.0 (3)	C34—C12—H12A	118.7
O13—Fe1—O13^ii^	111.0 (3)	C11—C12—H12A	118.7
O13^i^—Fe1—O1	107.9 (3)	C25—C13—C4	117.4 (14)
O13—Fe1—O1	107.9 (3)	C25—C13—H13A	121.3
O13^ii^—Fe1—O1	107.9 (3)	C4—C13—H13A	121.3
N6—Fe2—N3	93.0 (4)	N4—C14—C35	124.1 (18)
N6—Fe2—N1	93.4 (4)	N4—C14—H14A	118.0
N3—Fe2—N1	92.8 (4)	C35—C14—H14A	118.0
N6—Fe2—N5	82.3 (5)	C10—C15—C32	120.9 (16)
N3—Fe2—N5	174.4 (5)	C10—C15—C16	126 (2)
N1—Fe2—N5	90.5 (4)	C32—C15—C16	113 (2)
N6—Fe2—N2	174.4 (5)	C17—C16—C15	121 (2)
N3—Fe2—N2	92.1 (5)	C17—C16—H16A	119.7
N1—Fe2—N2	84.0 (4)	C15—C16—H16A	119.7
N5—Fe2—N2	92.7 (5)	C18—C17—C16	120 (2)
N6—Fe2—N4	93.3 (4)	C18—C17—H17A	119.9
N3—Fe2—N4	83.7 (5)	C16—C17—H17A	119.9
N1—Fe2—N4	172.6 (4)	C17—C18—C33	122 (2)
N5—Fe2—N4	93.5 (5)	C17—C18—C19	118 (2)
N2—Fe2—N4	89.6 (4)	C33—C18—C19	120 (2)
O3—W1—O4	104.9 (5)	C32—C19—N4	118.5 (13)
O3—W1—O7	102.9 (4)	C32—C19—C18	122.6 (18)
O4—W1—O7	86.4 (4)	N4—C19—C18	118.7 (17)
O3—W1—O12^ii^	95.3 (5)	C21—C20—N6	117.3 (11)
O4—W1—O12^ii^	159.7 (4)	C21—C20—C29	120.5 (12)
O7—W1—O12^ii^	87.9 (4)	N6—C20—C29	122.1 (11)
O3—W1—O12	97.3 (4)	N5—C21—C22	123.7 (13)
O4—W1—O12	90.0 (4)	N5—C21—C20	115.0 (11)
O7—W1—O12	159.7 (4)	C22—C21—C20	121.3 (13)
O12^ii^—W1—O12	88.6 (5)	C21—C22—C23	117.4 (16)
O3—W1—O1	163.4 (5)	C21—C22—C31	118.1 (15)
O4—W1—O1	88.2 (4)	C23—C22—C31	124.4 (16)
O7—W1—O1	87.8 (4)	C36—C23—C22	120.5 (15)
O12^ii^—W1—O1	72.1 (4)	C36—C23—H23A	119.7
O12—W1—O1	72.1 (4)	C22—C23—H23A	119.7
O5—W2—O7	103.3 (5)	N5—C24—C36	119.5 (17)
O5—W2—O9	101.8 (4)	N5—C24—H24A	120.3
O7—W2—O9	86.7 (4)	C36—C24—H24A	120.3
O5—W2—O14^ii^	97.7 (5)	C7—C25—C13	122.2 (14)
O7—W2—O14^ii^	92.2 (4)	C7—C25—H25A	118.9
O9—W2—O14^ii^	160.2 (4)	C13—C25—H25A	118.9
O5—W2—O11^ii^	95.9 (5)	N6—C26—C27	123.7 (13)
O7—W2—O11^ii^	160.7 (4)	N6—C26—H26A	118.2
O9—W2—O11^ii^	86.8 (4)	C27—C26—H26A	118.2
O14^ii^—W2—O11^ii^	87.8 (4)	C28—C27—C26	119.4 (15)
O5—W2—O13^ii^	166.2 (5)	C28—C27—H27A	120.3
O7—W2—O13^ii^	88.3 (4)	C26—C27—H27A	120.3
O9—W2—O13^ii^	86.2 (3)	C27—C28—C29	121.8 (13)
O14^ii^—W2—O13^ii^	74.0 (3)	C27—C28—H28A	119.1
O11^ii^—W2—O13^ii^	73.1 (3)	C29—C28—H28A	119.1
O6—W3—O2^ii^	103.8 (5)	C30—C29—C20	118.0 (14)
O6—W3—O2	102.5 (5)	C30—C29—C28	126.6 (14)
O2^ii^—W3—O2	85.6 (5)	C20—C29—C28	115.0 (12)
O6—W3—O11	96.5 (5)	C29—C30—C31	124.1 (16)
O2^ii^—W3—O11	159.7 (4)	C29—C30—H30A	117.9
O2—W3—O11	90.1 (4)	C31—C30—H30A	117.9
O6—W3—O10	96.9 (5)	C30—C31—C22	117.2 (14)
O2^ii^—W3—O10	90.0 (4)	C30—C31—H31A	121.4
O2—W3—O10	160.6 (4)	C22—C31—H31A	121.4
O11—W3—O10	87.5 (4)	C19—C32—C15	125.1 (16)
O6—W3—O13	165.8 (5)	C19—C32—N3	118.0 (15)
O2^ii^—W3—O13	87.1 (4)	C15—C32—N3	116.7 (16)
O2—W3—O13	87.2 (4)	C35—C33—C18	120.0 (17)
O11—W3—O13	73.0 (3)	C35—C33—H33A	120.0
O10—W3—O13	73.7 (4)	C18—C33—H33A	120.0
O8—W4—O9	103.7 (4)	C12—C34—C7	117.0 (15)
O8—W4—O4	104.3 (5)	C12—C34—H34A	121.5
O9—W4—O4	87.5 (4)	C7—C34—H34A	121.5
O8—W4—O10	95.1 (5)	C33—C35—C14	117.1 (18)
O9—W4—O10	90.8 (4)	C33—C35—H35A	121.5
O4—W4—O10	160.3 (4)	C14—C35—H35A	121.5
O8—W4—O14	96.0 (4)	C23—C36—C24	119.6 (17)
O9—W4—O14	160.3 (4)	C23—C36—H36A	120.2
O4—W4—O14	88.3 (4)	C24—C36—H36A	120.2
O10—W4—O14	86.8 (4)	C1—N1—C5	116.9 (10)
O8—W4—O13	164.7 (4)	C1—N1—Fe2	131.6 (8)
O9—W4—O13	87.6 (3)	C5—N1—Fe2	111.5 (8)
O4—W4—O13	86.2 (4)	C11—N2—C6	119.4 (12)
O10—W4—O13	74.2 (4)	C11—N2—Fe2	130.2 (10)
O14—W4—O13	72.9 (3)	C6—N2—Fe2	110.2 (8)
N1—C1—C2	124.0 (12)	C8—N3—C32	118.3 (13)
N1—C1—H1A	118.0	C8—N3—Fe2	132.7 (10)
C2—C1—H1A	118.0	C32—N3—Fe2	108.4 (10)
C3—C2—C1	121.8 (14)	C14—N4—C19	120.3 (13)
C3—C2—H2A	119.1	C14—N4—Fe2	128.8 (12)
C1—C2—H2A	119.1	C19—N4—Fe2	110.5 (10)
C2—C3—C4	116.7 (12)	C21—N5—C24	119.2 (13)
C2—C3—H3A	121.7	C21—N5—Fe2	113.5 (9)
C4—C3—H3A	121.7	C24—N5—Fe2	127.2 (11)
C5—C4—C13	120.5 (13)	C26—N6—C20	117.7 (11)
C5—C4—C3	117.5 (11)	C26—N6—Fe2	130.1 (9)
C13—C4—C3	122.0 (12)	C20—N6—Fe2	111.7 (8)
N1—C5—C4	123.0 (12)	Fe1—O1—W1^i^	119.9 (4)
N1—C5—C6	116.5 (11)	Fe1—O1—W1^ii^	119.9 (4)
C4—C5—C6	120.5 (12)	W1^i^—O1—W1^ii^	97.4 (5)
N2—C6—C5	117.7 (11)	Fe1—O1—W1	119.9 (4)
N2—C6—C7	123.9 (13)	W1^i^—O1—W1	97.4 (5)
C5—C6—C7	118.1 (13)	W1^ii^—O1—W1	97.4 (5)
C25—C7—C6	120.5 (14)	W3^i^—O2—W3	152.4 (5)
C25—C7—C34	124.2 (15)	W1—O4—W4	150.8 (5)
C6—C7—C34	115.3 (14)	W2—O7—W1	151.3 (5)
N3—C8—C9	121.7 (16)	W4—O9—W2	150.3 (5)
N3—C8—H8A	119.2	W4—O10—W3	115.8 (4)
C9—C8—H8A	119.2	W3—O11—W2^i^	116.6 (4)
C10—C9—C8	126.2 (19)	W1^i^—O12—W1	118.3 (5)
C10—C9—H9A	116.9	Fe1—O13—W2^i^	121.3 (4)
C8—C9—H9A	116.9	Fe1—O13—W4	120.8 (4)
C9—C10—C15	116.0 (18)	W2^i^—O13—W4	97.7 (3)
C9—C10—H10A	122.0	Fe1—O13—W3	118.3 (5)
C15—C10—H10A	122.0	W2^i^—O13—W3	97.2 (3)
N2—C11—C12	121.5 (14)	W4—O13—W3	96.0 (3)
N2—C11—H11A	119.2	W2^i^—O14—W4	115.2 (4)
C12—C11—H11A	119.2		

Symmetry codes: (i) −*y*+1, *x*−*y*, *z*; (ii) −*x*+*y*+1, −*x*+1, *z*.
